# Estimation of Several Wood Biomass Calorific Values from Their Proximate Analysis Based on Artificial Neural Networks

**DOI:** 10.3390/ma18143264

**Published:** 2025-07-10

**Authors:** I Ketut Gary Devara, Windy Ayu Lestari, Uma Maheshwera Reddy Paturi, Jun Hong Park, Nagireddy Gari Subba Reddy

**Affiliations:** 1 Department of Materials Engineering and Convergence Technology, Gyeongsang National University, Jinju 52828, Republic of Korea; garydevara@gnu.ac.kr (I.K.G.D.); lestariwa@gnu.ac.kr (W.A.L.); 2 Department of Mechanical Engineering, CVR College of Engineering, Hyderabad 501510, Telangana, India; maheshpaturi@cvr.ac.in; 3 School of Materials Science and Engineering, Engineering Research Institute, Gyeongsang National University, Jinju 52828, Republic of Korea

**Keywords:** Artificial Neural Networks, higher heating value, predictive model, proximate analysis, wood biomass

## Abstract

The accurate estimation of the higher heating value (HHV) of wood biomass is essential to evaluating the latter’s energy potential as a renewable energy material. This study proposes an Artificial Neural Network (ANN) model to predict the HHV by using proximate analysis parameters—moisture, volatile matter, ash, and fixed carbon. A dataset of 252 samples (177 for training and 75 for testing), sourced from the Phyllis database, which compiles the physicochemical properties of lignocellulosic biomass and related feedstocks, was used for model development. Various ANN architectures were explored, including one to three hidden layers with 1 to 20 neurons per layer. The best performance was achieved with the 4–11–11–11–1 architecture trained using the backpropagation algorithm, yielding an adjusted R^2^ of 0.967 with low mean absolute error (MAE) and root mean squared error (RMSE) values. A graphical user interface (GUI) was developed for real-time HHV prediction across diverse wood types. Furthermore, the model’s performance was benchmarked against 26 existing empirical and statistical models, and it outperformed them in terms of accuracy and generalization. This ANN-based tool offers a robust and accessible solution for carbon utilization strategies and the development of new energy storage material.

## 1. Introduction

Energy consumption has risen sharply in recent times, increasing the demand for fuels to satisfy the growing energy needs of humanity [1]. Due to the instability of oil prices, concerns about energy security, global warming, and future demands, fuel efficiency has become a top priority [2]. To address these issues, renewable energy sources that are eco-friendly and sustainable and produce lower emissions provide promising alternatives [3]. Among these, biomass offers significant potential as a renewable source for biofuel production, capable of substituting the limited fossil fuel reserves [4].

Nowadays, one of the most well-known energy sources is biomass. Biomass is a promising source of energy production to replace fossil fuels as a renewable energy source in various applications [1] and is converted into energy through mechanical–chemical processes such as gasification, pyrolysis, or combustion. Several studies have recently focused on the various aspects and outcomes of using biomass conversion [2] as a source of energy, including the development of an oxo synthesis plant [3], carbon monitoring [4], resource efficiency, and climate change mitigation [5], as well as the requirements of pyrolysis and combustion processes [6]. A comprehensive characterization of biomass is necessary to provide detailed information about its properties as a fuel, including thermal, chemical, and physical qualities [7,8,9]. Among the various properties, the relationship between chemical characteristics—particularly those derived from proximate and ultimate analyses—and the higher heating value is of critical importance, as it serves as a key indicator of the energy potential of solid biomass fuels [10,11,12]. The HHV represents the heat released during combustion, specifically generated when water vapor condenses into liquid [13]. There are three main types of models used to calculate the HHV of various biofuels, based on ultimate and proximate analyses, physical properties, and chemical composition [14,15]. Proximate analysis refers to the determination of moisture, ash, volatile matter, and fixed carbon (or char) content in a sample, typically expressed as a mass percentage [16]. This analysis provides a rapid and straightforward method to characterize the physical properties of biomass [17,18].

Several correlations have been proposed to estimate the HHV of biomass using proximate analysis data [8,17,18], and additional correlations have been developed to predict the ultimate analysis of biomass from proximate data [19]. However, these correlations often rely on linear or empirical relationships, which exhibit limited accuracy and may not effectively capture the nonlinear nature of biomass properties [20,21]. Furthermore, many of the correlations used in simulation programs, such as Aspen Plus, were originally developed for coal. As a result, applying them to solid biomass materials (e.g., wood and straw) can yield inaccurate results [22]. For instance, a study using the Aspen Plus V7.2 2010 simulator for simulating thermochemical conversion plants conducted by DBFZ Germany (Heidelberg, Germany, 2012) employed correlations developed for coal by Boie, Dulon, Moot–Spooner, Grummel–Davis, and the IGT. While these models produced estimates closer to experimental HHVs, significant deviations remained [17,23,24,25]. Moreover, such models are typically effective only within the range of the original experimental data and lack adaptability unless new equations are specifically derived [26,27]. Therefore, a more advanced approach is needed for accurately modeling nonlinear relationships and simplifying well to both existing and unseen data.

Artificial Neural Networks (ANNs), support vector machines (SVMs), random forest regression (RFR), and polynomials are among the nonlinear mathematical models created to predict the HHV [19,20,21]. The ANN has been reported as a suitable model for estimating the HHV and showed a high specific regression coefficient, high estimation, and a high degree of predicting the HHV [22,23]. Artificial Neural Networks (ANNs) are among the most widely developed models across various applications due to their high accuracy and adaptability, particularly when sufficient experimental data are available for training [24,25]. ANN models learn from predefined datasets by capturing complex nonlinear relationships between input (independent) and output (dependent) variables [26]. Each experiment contributes a single data point in the dataset, characterized by input features such as physicochemical properties and output values like HHVs. Increasing the number of high-quality data points can further enhance the performance of ANN models. When trained on consistent and reliable experimental data, ANNs can deliver repeatable and trustworthy predictions. Ultimately, the accuracy of ANN-based predictions depends heavily on the quality and clarity of the training and test datasets [27]. Several studies have demonstrated the effectiveness of ANN models in predicting the HHV of biomass. For example, Hosseinpour et al. [28] developed a neural network-adapted partial least squares model based on proximate analysis, resulting in user-friendly MATLAB software v.R2016a for HHV prediction. Uzun et al. [12] enhanced an ANN model by using proximate analysis data, achieving a high correlation coefficient and a low root mean square error (RMSE) in HHV prediction across various biomass samples. Similarly, Veza et al. [29] applied different ANN algorithms to predict the HHV, achieving high accuracy with elevated R^2^ values and reduced prediction errors, including mean absolute deviation (MAD), root mean square error (RMSE), and mean absolute percentage error (MAPE). However, ANN models lack limitation input diversity and, in comparison with other machine learning methods, explainability and interpretability.

To overcome these problems, an ANN model with explainability and interpretability that can support decision making in applications related to the combustion behavior of biomass is needed. Therefore, this research study has the goal to develop a GUI-based ANN model for the rapid and accurate prediction of HHVs based on the physical properties of various solid biomass types. The objective of this research study is to enhance the explainability of biomass modeling by analyzing input features using scatter matrix plots and Pearson correlation coefficients. Additionally, this study investigates how variations in ANN model parameters influence prediction accuracy, offering insights into the model’s robustness and reliability.

## 2. Materials and Methods

### 2.1. Data Collection

The dataset used in this study was sourced from the Phyllis database (https:/Phyllis.nl, accessed on 4 March 2025), maintained by TNO Biobased and Circular Technologies. While the Phyllis contains comprehensive physicochemical data for a wide range of biomass types, including algae, biochar, and biogas feedstocks, for this study, we specifically restricted the dataset to wood-based biomass, extracting 252 data records [30]. Since extracting and organizing these data from the online database is a labor-intensive process, the complete dataset is provided in Table S1 to support future research and reproducibility. To ensure model relevance and consistency in HHV prediction, 177 samples were used for training and 75 for testing the ANN model. The input variables were physical characteristics such as moisture (M), ash (A), volatile matter (VM), and fixed carbon (FC). The output variable was the HHV. Scatter matrix plots show the relationship between them or how much individual physical variables are affected by another variable.

**Figure 1:**
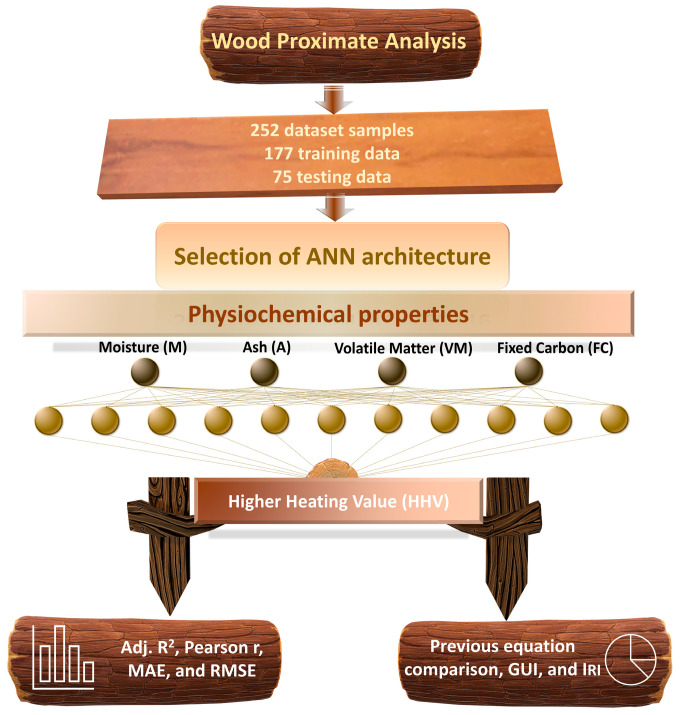
Flowchart of ANN-based prediction system using wood proximate analysis data.

The schematic process of the ANN-based prediction system designed to use proximate analysis data to estimate the HHV of wood biomass is shown in Figure 1. The proposed methodology begins with wood proximate analysis, where four physiochemical properties—moisture (M), ash (A), volatile matter (VM), and fixed carbon (FC)—are extracted as model inputs. These are fed into an Artificial Neural Network (ANN) with a carefully selected architecture, consisting of three hidden layers with 11 neurons each (4–11–11–11–1 structure). The multi-layer structure enables the model to learn not only complex interactions but also nonlinear interactions between input variables and HHVs. During training, the backpropagation algorithm adjusts the connection weights to minimize prediction error. The model is validated using performance metrics such as adjusted R^2^, Pearson r, MAE, and RMSE. The final trained model achieves high predictive accuracy and generalization performance. To enhance accessibility, a graphical user interface (GUI) was developed, and the ANN model’s output was benchmarked against 26 existing equations. This workflow illustrates a comprehensive pipeline from raw input data to reliable, real-time HHV predictions, with clear potential for energy system optimization and carbon reduction strategies. This makes the model easier to understand and apply in real-world scenarios. 

**Figure 2:**
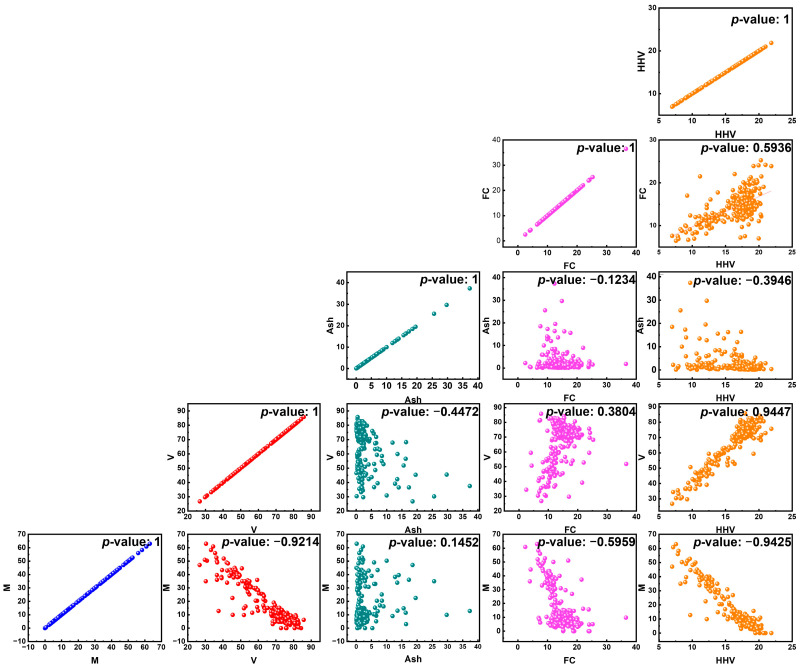
Visual correlation analysis of physicochemical properties and higher heating value.

To investigate the correlations among the parameters, we plot each parameter in a linear graph. Figure 2 shows the correlation between input parameters and output HHVs for several kinds of biomass. There is a complex correlation between physical properties and the HHV of biomass. The linear correlation between two features can be extracted from the Pearson correlation coefficient within the range 1 to −1. The pairwise scatter plot matrix in this figure shows the relationships between the higher heating value of the samples and their main physicochemical characteristics, such as moisture content, volatile matter, ash content, fixed carbon, and moisture content. While the off-diagonal scatter plots illustrate the bivariate associations between variables and their accompanying Pearson correlation coefficients (referred to as the “*p*-value” in this context), the diagonal panels display the distribution of each variable. The relationships between M and HHV (*p*-value ≈ 0.842), between FC and HHV (*p*-value ≈ 0.836), and between M and V (*p*-value ≈ 1) are all strongly positive. In contrast, a strong negative association between HHV and ash concentration (*p*-value = −0.856) suggests that more ash lowers the heating potential. There are less strong relationships between M and FC and between V and ash. The physicochemical characteristics that have the greatest impact on the HHV may be understood by these visual insights, which will direct future model development and optimization.

**Figure 3:**
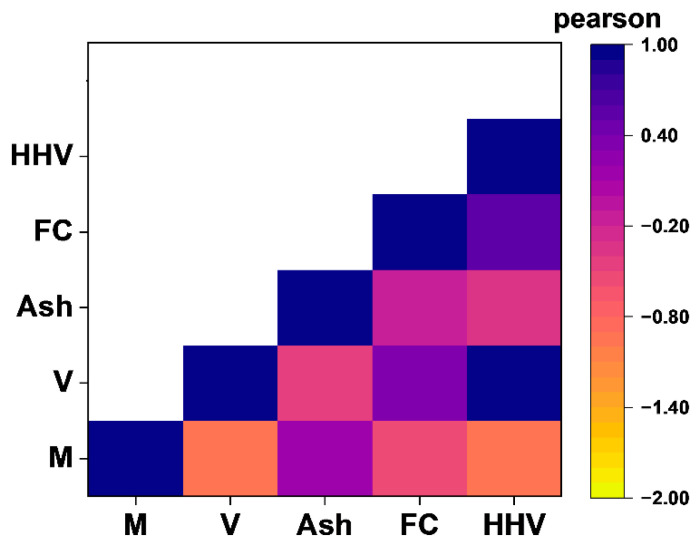
Correlation heat map of physicochemical parameters.

The heat map plot of the Pearson correlation is plotted to simply investigate the correlations among the parameters. The Pearson correlation heat map is shown in Figure 3. The correlation coefficient quantifies the strength and direction of a linear relationship between two variables. A **positive coefficient** indicates that both variables tend to increase together, while a **negative coefficient** implies that as one variable increases, the other decreases. Coefficients near **zero** suggest a weak or negligible linear relationship, meaning changes in one variable are not strongly associated with changes in the other.

The pairwise correlations among five physicochemical parameters, moisture content, volatile matter, ash content, fixed carbon, and the higher heating value, are shown in this heat map. From −1 (strong negative correlation, represented by orange) to +1 (strong positive correlation, represented by dark blue), the color gradient indicates the association’s intensity and direction.

Several important discoveries are highlighted in the heat map. Both moisture content and ash content show a strong negative association with the HHV, suggesting that higher concentrations of these elements lower the biomass’s potential for energy production. However, the HHV and fixed carbon have a high positive association, confirming FC’s position as the main source of fuel energy. Furthermore, the HHV and volatile matter have a relatively positive correlation, but ash content and volatile matter have a negative correlation. Moisture and volatile matter have a significant inverse correlation, which emphasizes their close composition. This graphic analysis provides a clear and understandable synopsis of feature interdependencies, which enhances the scatter plot matrix results. The selection of features and the understanding of the model in later predictive analyses are greatly aided by these relationships.

### 2.2. Development of GUI-Based ANN Model and Evaluation Procedure

The accuracy and reliability of the ANN model are strongly influenced by the quality of the input variables. High-quality, relevant inputs enable the model to learn meaningful patterns, while poor or noisy data can reduce predictive performance and generalization ability.

Table S1 shows the 252 dataset samples utilized to create the ANN models. The data were divided into a 177-sample training dataset for model development and a 75-sample test dataset to evaluate the developed model. All the process parameters were normalized between 0.1 and 0.9, and the detailed explanation and equations for the normalization process were presented in previous reports [31].

The ANN model in this study was developed using the backpropagation learning algorithm, which updates weights by propagating the error backward through the network. A sigmoid activation function was adopted to introduce nonlinearity into the learning process, enabling the model to capture complex relationships between input and output variables. The training algorithm and detailed procedure closely follow those reported in earlier works [32,33]. For implementation, both C language and JAVA were employed to develop the core ANN engine and a user-friendly graphical user interface (GUI), allowing users to operate the model without requiring programming expertise. The final ANN configuration consists of four input nodes representing the selected input features and one output node corresponding to the predicted property.

## 3. Results

### 3.1. Neural Network Architecture Optimization

The architecture of the ANN model was optimized through a systematic evaluation of various configurations. Initially, the number of **hidden layers** was varied from one to three, and for each case, the number of **neurons in each hidden layer** was adjusted from 1 to 20. These trials were conducted under fixed values of **learning rate**, **momentum coefficient**, and **number of training iterations**, as illustrated in Figure S1. After determining a promising baseline structure, additional experiments were performed by individually tuning the **learning rate**, **momentum**, and **iterations** to further refine the model’s performance. The effectiveness of each configuration was assessed using standard evaluation metrics such as the **root mean square error (RMSE)**, the **mean absolute error (MAE)**, and the **coefficient of determination (R^2^)** on the test data. The final architecture was selected based on the best trade-off between prediction accuracy and model stability.

**Figure 4:**
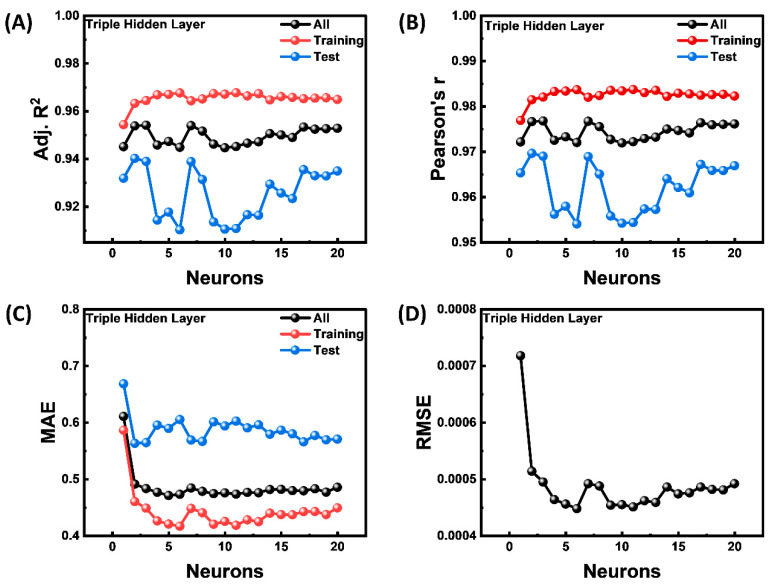
Performance metrics of a triple-hidden-layer neural network with varying neuron counts for all data, and training, and test datasets. (**A**) Adj. R^2^, (**B**) Pearson r, (**C**) MAE, and (**D**) RMSE.

The architecture with three hidden layers outperformed both the single-layer and two-layer configurations. The best model by varying the hidden neuron number and layers is provided in Figure 4. The training curves in Figure 4A and Figure 4B display high and consistent values of more than 0.96 and 0.98, respectively, for every neuron configuration, suggesting strong fitting capability. However, as the number of neurons increases, the test set performance deteriorates and becomes unstable, especially when there are more than three to five neurons per layer. The model’s training performance remains high, but test performance degrades. This divergence is a classic sign of overfitting, where the model captures noise or specific patterns in the training data that do not generalize well [22,32]. The model picks up noise or patterns unique to the training set that are difficult to apply to fresh data. Figure 4C supports this pattern substantially. The training set’s MAE continuously drops as more neurons are added, indicating better training data memorization. But without a discernible decrease, the test MAE continuously rises, highlighting the merger advantage of more complicated models for unknown data. Figure 4D shows a similar pattern, where the error decreases initially as the number of neurons increases up to three–five, after which additional additions result in diminishing returns. The RMSE values show a slight improvement as the number of neurons increases, stabilizing at around 0.00045. Based on these results, we selected a model with a number of neurons of 11, which provides a balance between underfitting and overfitting. This configuration ensures robust performance on both the training and test datasets.

**Figure 5:**
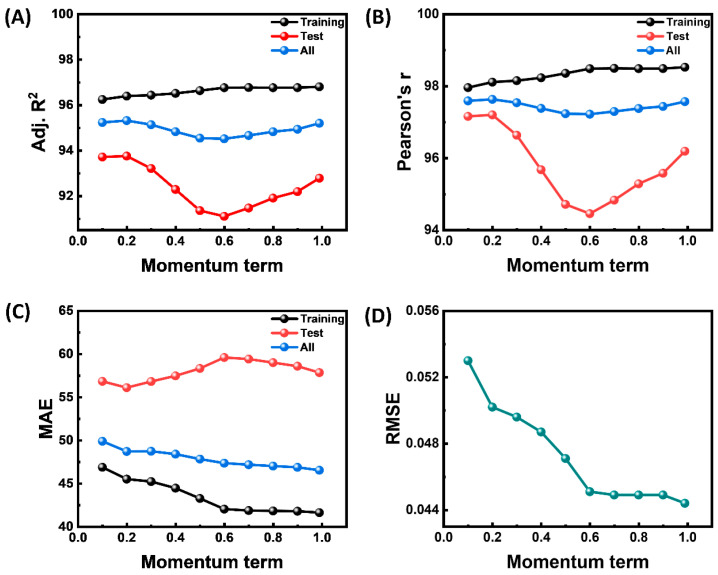
Effect of momentum term on performance of ANN model. (**A**) Adj R^2^, (**B**) Pearson r, (**C**) MAE for all data, and training, and test datasets; (**D**) RSME.

The suggested predictive models’ key performance indicators are guided by the optimizer’s momentum term (0.1–1.0), as shown in Figure 5. The training curves (gray) for adjusted R^2^ and Pearson r (Figure 5A,B) climb monotonically as the momentum increases, while MAE steadily decreases (Figure 5C). This confirms that more momentum speeds up convergence and enables the network to capture more of the signal seen in the training data. However, the test set curves (red) show a shallow but consistent U or inverted U pattern: generalization deteriorates when momentum is in the mid-range (0.4–0.6), reaching its lowest values for all four metrics around 0.55, and then recovers as the optimizer becomes highly inertial (≥0.8). The RMSE plot in Figure 5D exhibits the same pattern, with the error decreasing dramatically until momentum ≆0.6 and then plateauing. These results collectively show a clear trade-off: very high momentum reduces extreme errors (RMSE) at the expense of slightly higher average errors (MAE) and a higher risk of overshooting during training, while low to moderate momentum (0.1–0.3) provides the most stable balance between bias and variance. Choosing values outside of the 0.4–0.6 range produces models that generalize considerably better and are therefore more appropriate for deployment in the intended application. The brief performance decrease with mid-range momentum highlights the significance of appropriately adjusting this hyperparameter.

**Figure 6:**
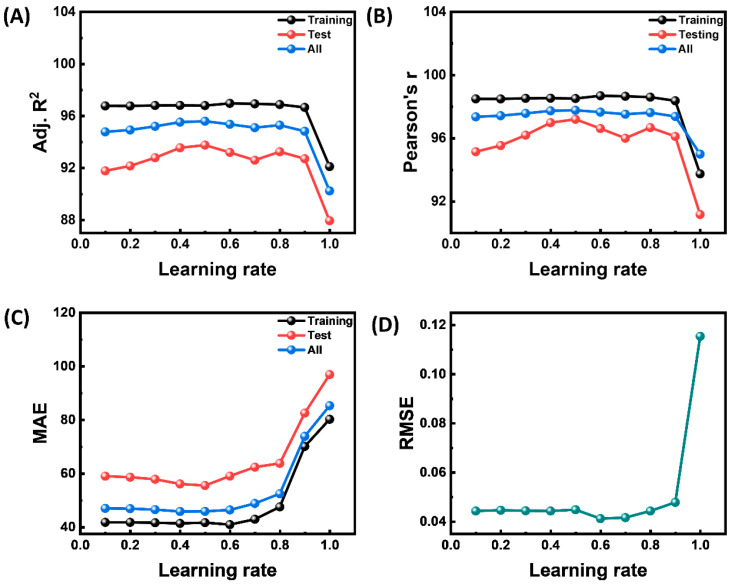
Effect of learning rate on performance of ANN model. (**A**) Adj R^2^, (**B**) Pearson r, (**C**) MAE for all data, and training, and test datasets; (**D**) RSME.

In Figure 6, the model’s sensitivity to the learning rate (0–1.0) is shown for four complementary performance criteria. As the step size increases, the optimizer can still fit the training data accurately because the training curves for modified R^2^ and Pearson r (Figure 6A,B) stay almost flat and high (≈98%) up to a learning rate of 0.8. The test set curves (red) show a steady improvement from low rates, peaking between 0.4 and 0.6, and then a sudden fall when the rate surpasses 0.9. This indicates that excessively aggressive updates destabilize convergence and weaken explanatory power on unseen samples. In contrast, generalization is more discriminating. While the RMSE for the external validation set remains flat at ≈0.042 until it spikes three times at a learning rate of 1.0, the MAE for the test set achieves its minimum near 0.5–0.6 and increases fast beyond 0.8. These error metrics (Figure 6C,D) follow the same pattern. These results collectively point to a broad optimum around learning rates of 0.4–0.6, where the bias-adjusted goodness of fit and error statistics are simultaneously maximized. They also warn that settings over 0.8 cause significant divergence and should be avoided in production deployments. In addition, the effect of iteration on the performance of the ANN model is explained in Figure S2. The most effective ANN model is reached at 10,000 iterations.

### 3.2. Transformations of Synaptic Weights

**Figure 7:**
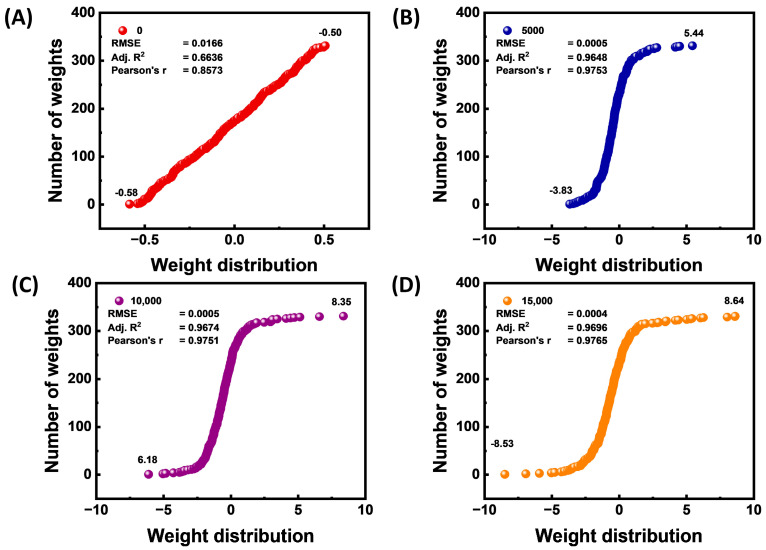
ANN weight distribution in different training stages. (**A**) 0, (**B**) 5000, (**C**) 10,000, and (**D**) 15,000 iterations.

The network weight landscape’s evolution from random initialization to full convergence is depicted in Figure 7, which also connects this structural evolution to predictive accuracy. A characteristic of ignorant random seeding that is associated with poor performance (Adj. R^2^ ≈ 0.66; RMSE ≈ 1.7 × 10^−2^) is weights that are symmetrically and tightly distributed about zero (−0.6 to +0.6) at iteration 0, as shown in Figure 7A. The distribution expands by almost an order of magnitude (−3.8 to +5.4) and takes on a sigmoidal shape after just 5000 iterations (Figure 7B). This expansion is accompanied by a two order of magnitude decrease in error (RMSE ≈ 5 × 10^−4^) and an increase in explanatory power (Adj. R^2^ ≈ 0.96). Most parameters attain near-optimal magnitudes by the time the curve steepens, and its support stabilizes (6.2 to +8.4) at 10,000 iterations (Figure 7C). An additional 15,000 steps (Figure 7D) result in a small performance boost and just slightly wider tails (−8.5 to +8.6), suggesting diminishing benefits. When combined, these snapshots show an early, clear polarization of weights that supports the sharp performance boost seen in earlier figures. They also support the use of an early-stopping criterion when the sigmoidal weight profile and hence the predictive plateau has been reached. In addition, the magnitude and direction of network weights distribution at different iteration rates, 20,000, 25,000, and 30,000, are provided as shown in Figure S3.

### 3.3. Index of Relative Performance

**Figure 8:**
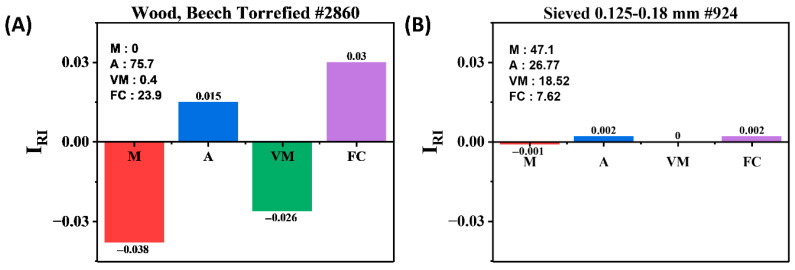
Relative importance index of physicochemical variables: (**A**) high HHV and (**B**) low HHV.

To gain insights into the relative influence of each input feature, Pearson correlation and the relative importance index (I_RI_) were applied. These statistical methods help interpret which variables most strongly impact the model’s output, offering guidance on the role of physiochemical properties in determining the HHV. However, it is important to note that these approaches do not constitute explainability in the formal sense used in interpretable machine learning (IML), where methods such as SHAP or LIME are typically employed [33,34,35]. Therefore, our use of the I_RI_ and Pearson correlation should be regarded as tools for enhancing interpretability, not complete explainability. The estimated HHV using the relative importance index (I_RI_) for two representative biomass samples is shown in Figure 8 and Figure S4 concerning four physicochemical characteristics: moisture content, ash content, volatile matter, and fixed carbon. The biomass in Figure 8A has a high HHV (wood, beech torrefied #2860; Table S1), whereas the biomass in Figure 8B has a low HHV (sieved 0.125–0.18 mm #924).

Fixed carbon shows the most positive contribution to the HHV (IRI = 0.03) in the high-HHV sample (Figure 8A), which is consistent with its direct energetic value. Even though the proportion of ash content is considerable (75.7%), it exhibits a small positive IRI (0.015), which could indicate model compensation or a confounding influence. The energy needed for water evaporation, on the other hand, causes the moisture content to have a significant negative impact (I_RI_ = −0.038), confirming its negative influence on fuel efficiency. A slight negative influence is also seen from VM (IRI = −0.026).

All factors show nearly negative IRI values for the low-HHV sample (Figure 8B), indicating that individual traits do not affect the HHV in this sample. While ash and fixed carbon both show a modestly positive contribution (I_RI_ = 0.002), moisture content significantly lowers the HHV (I_RI_ = −0.001). A more homogeneous or restricted composition among low-HHV biomass classes may be the cause of the absence of prominent predictors.

### 3.4. Creation of Virtual Biomass HHV System

**Figure 9:**
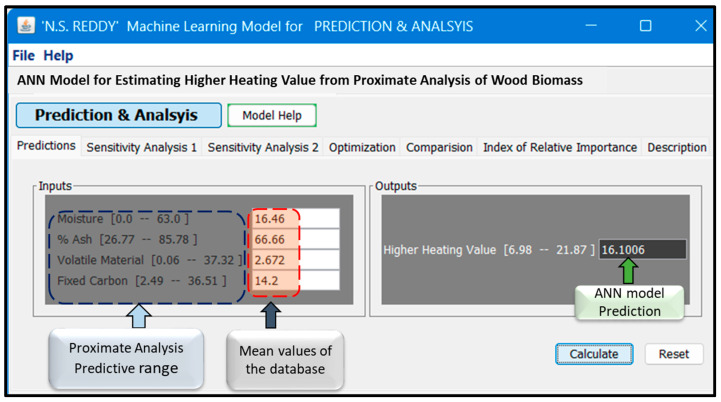
Graphical user interface (GUI) of our Artificial Neural Network model.

Figure 9 illustrates the custom GUI developed to estimate the HHV of wood biomass by using an ANN model trained on proximate analysis data. This interface, developed as part of a nonlinear system modeling suite, offers a user-friendly platform for interacting with the prediction and the interface features.

**Input Panel (Left):** Users can enter or adjust key biomass properties—moisture content, volatile matter, ash content, and fixed carbon—either manually or by exploring the full predictive range derived from our database.

**Output Panel (Right):** The ANN computes and displays the predicted HHV. In the example screenshot, using the mean proximate analysis values (“virtual wood”), the model predicts an HHV of 16.1006 MJ/kg.

This GUI is powered by the synaptic weights of the ANN model, enabling highly accurate predictions for infinite combinations of input parameters within the learned domain. The model was trained on a diverse dataset, making it robust and efficient for predicting the HHV across a wide variety of wood types, including hypothetical or new biomass compositions.

Numerous linear, nonlinear, and polynomial equations have been proposed in the literature to describe the relationship between proximate analysis parameters and HHVs. This ANN-based model offers a unique, data-driven solution with superior accuracy and generalization capability. It is particularly well suited for rapid screening and decision making in biomass energy applications. The GUI is potentially applicable in some industries, such as energy [36] and biomass [37] industries, and in research institutions [38]. The GUI provides a fast and user-friendly platform for estimating the HHV of various wood biomass types, which can be crucial to fuel grading and procurement decisions in biomass-based power plants. By integrating the ANN model into operational workflows, companies can rapidly screen biomass feedstocks and make data-driven choices to optimize combustion efficiency and reduce carbon emissions. The GUI is a predictive tool for hypothesis testing and simulation not only for energy industry, reducing the need for extensive experimental calorimetry in research institutes.

### 3.5. Comparison of ANN Model Predictions for Biomass HHV with Experimental Results from the Literature and Proximate Analysis Data

**Figure 10:**
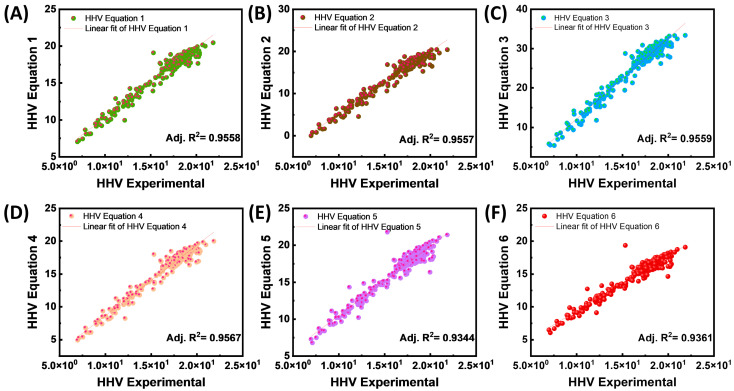
Comparison of experimental and predicted HHVs using proposed models based on (**A**) Equation (1), (**B**) Equation (2), (**C**) Equation (3), (**D**) Equation (4), (**E**) Equation (5), and (**F**) Equation (6).

A comparison between the HHVs predicted by the suggested machine learning models and empirically measured values is shown in Figure 10 for six distinct datasets: Equation (1) (Figure 10A), Equation (2) (Figure 10B), Equation (3) (Figure 10C), Equation (4) (Figure 10D), Equation (5) (Figure 10E), and Equation (6) (Figure 10F). The existing equations used based on proximate analysis are provided for comparison as shown in Table 1.

In addition, another 20 empirical formulas (Table S2) are provided to show a comprehensive comparison between experimental results and HHV equations, as shown in Figure S5. A fitted regression line and the associated coefficient of determination (R^2^), which measures the model’s prediction ability, are shown in each subplot together with the data distribution. The Equation (3) dataset produced the highest R^2^ value (0.9559), followed by Equation (1) (0.9558), Equation (2) (0.9557), and Equation (4) (0.9567), indicating that the models consistently performed well across most datasets. The robustness of the suggested modeling approach is demonstrated by these strong R^2^ values, which show great agreement between anticipated and experimental results. The models showed good prediction accuracy; however, the Equation (5) (0.9344) and Equation (6) (0.9361) datasets showed slightly lower R^2^ values.

**Figure 11:**
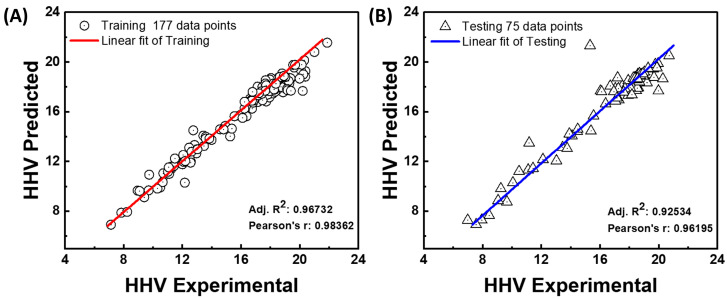
Correlation analysis between the experimental and predicted HHVs for (**A**) training and (**B**) test data points.

The correlation analysis between the experimental and anticipated HHVs for the training and test datasets is shown in Figure 11. The model’s performance on the test dataset (75 data points) is depicted in the right panel, and the model’s performance on the training dataset (177 data points) is displayed in the left panel. The Pearson correlation coefficient (r = 0.98362) and the adjusted coefficient of determination (Adj. R^2^ = 0.96732) are high, indicating a significant linear connection between the experimental and predicted HHVs throughout the training period, as shown in Figure 11A. It is confirmed that the model learned from the training data accurately according to the data points’ close alignment with the red linear fit line.

In the test phase (Figure 11B), the model continues to perform well in terms of prediction, with an adjusted R^2^ of 0.92534 and a Pearson r of 0.96195. Even though the test data exhibit somewhat more variability than the training data, the linear trend (blue line) is still strong, suggesting that the model can generalize well to new data. The constructed model can reliably and accurately predict HHVs across training and test sets, as these data collectively demonstrate.

## 4. Discussion

### 4.1. Comparison with Past Research

The model’s performance was measured against 26 other empirical and statistical models, and it outperformed them in terms of accuracy and generalization. Empirical formulas are supplied to demonstrate a full comparison between the HHV equations and the HHVs obtained via the ANN model. Following a statistical analysis of the factors required to develop the machine learning models, the HHV and Adj. R^2^ from proximate analysis data biomass were determined. Figure 10, Figure 11 and Figure S5 show the results of the above values. After performing calculations on 252 data, it is possible to infer that there is significant variability in the results. The average HHV in this study is 16.1 MJ/kg, with an Adj. R^2^ of 0.967, which is higher than the reported values in the Adj. R^2^ range of 0.54–0.95 [44,45,46]. In addition, the proposed HHV based on equations showed the Adj. R^2^ values range of 0.1557–0.9567. This finding is consistent with prior studies that found the ANN to be the best model for predicting the value of the HHV variable [19,47].

### 4.2. Research Implications

In accordance with the statistical metrics employed, the ANN model has optimal efficiency, indicating good forecast precision and dependability. Hyperparameters such as the number of hidden layers, momentum, learning rate, and training iterations are changed to optimize the architecture, resulting in a high level of accuracy and a smaller modeling error. An experiment was carried out by selecting input data fed into the ANN model. The use of the machine learning model in HHV biomass estimation demonstrated definite benefits in terms of prediction accuracy and dependability as measured by performance metrics including modified Adj. R^2^, Pearson correlation coefficient, MAE, and RMSE. The ANN model displayed lower RMSE and MAE values, along with high Adj. R^2^ values > 0.92 and a high Pearson coefficient > 0.95. This result is consistent with studies showing that ANNs frequently obtain high Adj. R^2^ values and lower RMSE and MAE values in estimation tasks by managing the data [19,22,48]. Furthermore, to predict the HHV in real time across various wood types, a GUI was created. The ANN model’s synaptic weights power this GUI with incredibly precise predictions for an endless number of input parameter combinations inside the learned domain. An HHV of 16.1006 MJ/kg was predicted by the model using the mean proximate analysis values, or “virtual wood.” This ANN-based GUI model offers a unique, data-driven solution with superior accuracy and generalization capability. It is particularly well suited for rapid screening and decision making in biomass energy applications [2,49].

## 5. Conclusions

The HHV of wood biomass was accurately predicted using proximate analysis parameters—moisture, volatile matter, fixed carbon, and ash content—via an Artificial Neural Network (ANN) model. A total of 252 data samples were analyzed, with 177 used for training and 75 for testing. The optimized network architecture (4–11–11–11–1), along with finetuned learning rate, momentum, and iteration count, yielded strong predictive performance, achieving adjusted R^2^ and Pearson r values consistently above 0.96 in training and above 0.92 in testing. Fixed carbon and volatile matter positively influenced the HHV, whereas moisture and ash had negative effects. Comparative evaluation showed that three hidden layers outperformed shallower configurations. To enhance usability, a graphical user interface (GUI) was developed for practical and rapid HHV prediction. The ANN model outperformed 26 existing empirical models and proved to be a robust, generalizable tool for biomass energy assessment, supporting low carbon technologies and green development initiatives.

To enhance accessibility, a user–friendly graphical interface was developed for real-time HHV prediction across diverse wood biomass types. Beyond prediction accuracy, the model has practical implications for promoting energy efficiency and carbon reduction: By enabling more precise selection and utilization of high-HHV biomass, it supports more efficient combustion system design and minimizes incomplete combustion, thereby reducing CO_2_ emissions. Although exact emission reductions depend on specific applications, improved HHV estimation can contribute to optimizing bioenergy systems, enhancing fuel economy, and supporting broader green development and low-carbon energy strategies. The ANN model also outperformed 26 existing empirical models, demonstrating its value as a robust and generalizable tool for biomass energy assessment. This ANN-based GUI model provides a distinctive, data-driven approach with enhanced accuracy and generalization potential. It is especially well suited for quick screening and decision–making in biomass energy applications.

## Figures and Tables

**Table 1:** The empirical formulas derived from the mentioned literature using data from proximate analysis.

Equation Number	Equations	Units	Ref.
(1)	HHV = 0.1905 × VM + 0.2521 × FC	(MJ/kg)	[39]
(2)	HHV = −10.81408 + 0.3133 × (VM + FC)	(MJ/kg)	[40]
(3)	HHV = 0.03 × Ash − 0.11 × M + 0.33 × VM + 0.35 × FC	(MJ/kg)	[41]
(4)	HHV = 3.0368 + 0.2218 × VM + 0.2601 × FC	(MJ/kg)	[14]
(5)	HHV = 0.3543 × FC + 0.1708 × VM	(MJ/kg)	[42]
(6)	HHV = 0.312 × FC + 0.1534 × VM	(MJ/kg)	[43]

HHV: higher heating value; VM: volatile matter; FC: fixed carbon.

## Data Availability

The original contributions presented in this study are included in the article/Supplementary Materials. Further inquiries can be directed to the corresponding authors.

## Supplementary Materials

The following supporting information can be downloaded at: https://www.mdpi.com/article/10.3390/ma18143264/s1. Figure S1: Comprehensive performance analysis of ANN model across different neuron counts and layers. The models were used with 1, 2, and 3 layers with 1 to 20 neurons each. Adj R2 for (A) all data, and (B) test, and (C) training datasets. Pearson for (D) all data, and (E) test, and (F) training datasets. MAE for (G) all data, and (H) training, and (I) test datasets. (J) RSME for 1-, 2-, and 3-layer ANN models; Figure S2: Effect of iteration on performance of ANN model. (A) Adj R^2^, (B) Pearson r, (C) MAE for all data, and training, and test datasets; (D) RSME; Figure S3: ANN weight distribution in different training stages. (A) 20000, (B) 25000, and (C) 30000 iterations; Figure S4: Relative importance index of physicochemical variables: (A–C) high higher heating values and (D–F) low higher heating values; Figure S5: Comparison of experimental and predicted HHVs using proposed models: (A) Equation (7), (B) Equation (8), (C) Equation (9), (D) Equation (10), (E) Equation (11), (F) Equation (12), (G) Equation (13), (H) Equation (14), (I) Equation (15), (J) Equation (16), (K) Equation (17), (L) Equation (18), (M) Equation (19), (N) Equation (20), (O) Equation (21), (P) Equation (22), (Q) Equation (23), (R) Equation (24), (S) Equation (25), and (T) Equation (26); Table S1: The empirical formulas derived from the mentioned literature using data from proximate analysis; Table S2: Raw data used to generate the ANN proposed empirical equations [14,41,43,44,45,50,51,52,53,54,55,56,57].
