# Supplementary material for: Estimation of Several Wood Biomass Calorific Values from Their Proximate Analysis Based on Artificial Neural Networks
Source: Materials (Basel). 2025 Jul 10;18(14):3264. doi: 10.3390/ma18143264 (PMC12298734; doi:10.3390/ma18143264)
Supplement: Supplementary file 1 [file materials-18-03264-s001.zip › materials-3699676-supplementary.pdf]

Supplementary Materials

Estimation of Several Wood Biomass Calorific Values from Their Proximate Analysis Based on Artificial Neural Networks

I Ketut Gary Devara [1,†], Windy Ayu Lestari [1,†], Uma Maheshwera Reddy Paturi [2], Jun Hong Park [1,*] and Nagireddy Gari Subba Reddy [3,*]

[1] Department of Materials Engineering and Convergence Technology, Gyeongsang National University, Jinju 52828, Republic of Korea

[2] Department of Mechanical Engineering, CVR College of Engineering, Hyderabad 501510, Telangana, India

[3] School of Materials Science and Engineering, Engineering Research Institute, Gyeongsang National University, Jinju 52828, Republic of Korea

* Correspondence: yakte@gnu.ac.kr (J.H.P.); nsreddy@gnu.ac.kr (N.G.S.R.)

† These authors contributed equally to this work.

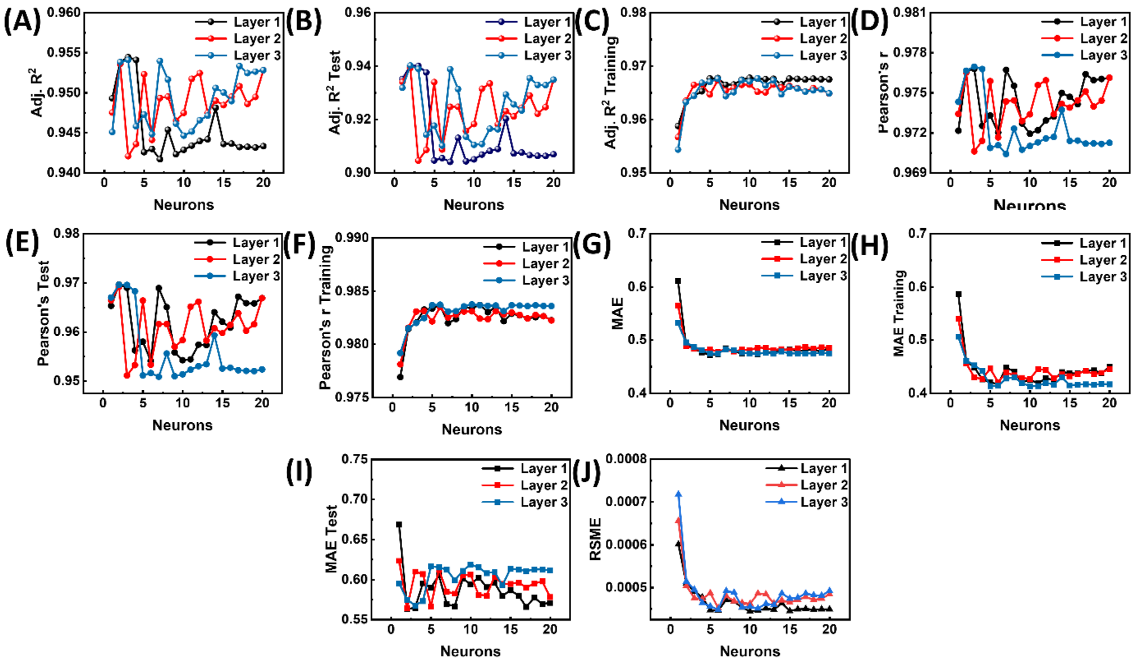

Figure S1. Comprehensive performance analysis of ANN models across different neuron counts and layers. The model was used for 1, 2, and 3 layers within 1 to 20 neurons. Adj R^2 for (**A**) all, (**B**) testing, and (**C**) training datasets. Pearson's for (**D**) all, (**E**) testing, and (**F**) training datasets. MAE for (**G**) all, (**H**) training, and (**I**) testing datasets. (**J**) RSME for 1, 2, and 3-layer ANN models.

Figure S1 provides a thorough assessment of how neuron counts and hidden layer depth (1, 2, and 3-layer) affect an ANN's ability to forecast the HHV of wood-based biomass based on proximate analysis data. A few statistical metrics are used to evaluate the model's performance, including MSE, Pearson correlation coefficient, R-squared (Adj. R^2), and MAE across the various dataset divisions (all, test, training). Each sub-figure represents a distinct statistic that sheds light on the correctness and stability of the model across different topologies. The R^2 values computed using the complete dataset are displayed by RSQ All. As the number of neurons increases, the performance of the single hidden layer model remains comparatively steady, while deeper structures introduce more variance,

as shown in Figure S1A. The R^2 values for the test subset are shown in Figure S1B, Adj. R^2 test. The findings show the possibility of overfitting, as they vary more dramatically across neuron counts, particularly in deeper networks. Figure S1C depicts that the training data regularly displays excellent R^2 values, especially for 1-layer and 2-layer models, suggesting robust fitting capabilities. Figure S1D shows the correlation between Pearson's r and the number of neurons in a single hidden layer, fluctuating and increasing as the number of neurons rises, occupying a closed value around 0.975 with layer 2 at 20 neurons. In addition, Pearson's r test shows that the number of neurons in a network rises above a particular threshold; correlation values on the test dataset indicate increased instability in deeper networks, indicating reduced generalizability, as shown in Figure S1E. Besides that, Figure S1F training calculated in Pearson's r training data regularly has high Pearson coefficients (above 0.98), which strengthens the model's ability to learn from the training set. Furthermore, the average value has been demonstrated in three categories: all, training, and test datasets. Figure S1G shows single-layer models with more than ten neurons stabilize at about 0.47, the lowest inaccuracy. Figure S1H displays mistake patterns when training. Once more, error diminishes as the number of neurons increases, and layers 1 and 2 perform better than layer 3, while Figure S1I shows Moderate-neuron shallow networks exhibiting reduced error values and greater stability. Model error can be determined using the MSE measure. With an increase in neurons, the MSE dramatically drops and stabilizes for all layer configurations above 8–10 neurons, as demonstrated in Figure S1J. The developed ANN model has 252 datasets associated with weights and holds information about the relationship between physicochemical variables and higher heating value. The optimum network architecture obtained was 4-11-11-11-1 by varying hidden layers, neurons in the hidden layer, and iterations. This optimum network architecture value was the basis for building the performance of ANN models by varying the momentum terms, learning rates, and iterations.

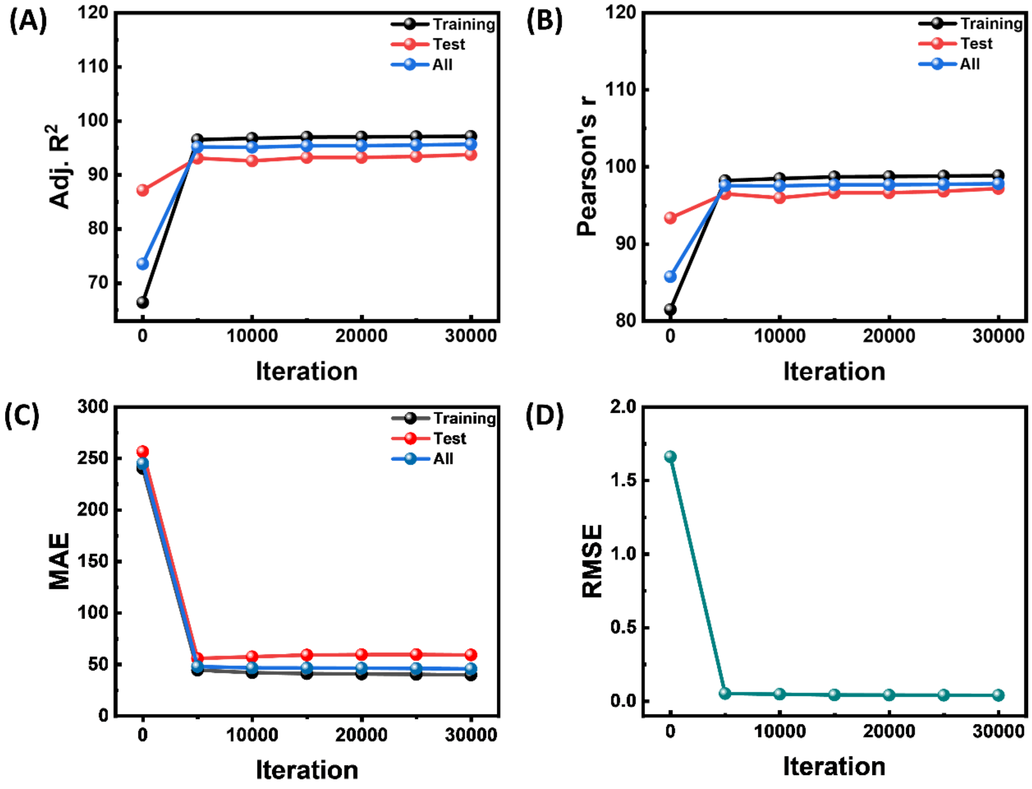

Figure S2. Effect of iteration on the performance of ANN models. (**A**) Adj R^2, (**B**) Pearson's r, (**C**) MAE for all, training, and testing datasets, and (**D**) RSME.

Figure S2 shows the evolution of each key performance indicator from a random start (iteration 0) to 30,000 optimization steps during training. All four panels exhibit the same distinctive pattern: a protracted but steady plateau after a quick convergence during the first ≆5,000 repetitions. Pearson's r (Figure S2A and S2B) and adjusted R^2 both increase quickly to between 96 and 98 percent for both training and test sets, then drop by less than one percentage point, suggesting that the network captures nearly all learnable variance at an early stage. The error metrics (Figure S2C and S2D) also show that over-fitting is not present, as they collapse from large initial values (MAE > 250; RMSE ≈ 1.6) to their minimal (MAE ≈ 45–55; RMSE ≈ 0.02) within the same window and then remain essentially flat, with training and test-set curves overlapping throughout. These trajectories demonstrate that the model achieves its ideal bias-variance balance well before 10,000 iterations; more training produces no discernible improvements, proving the effectiveness and scientific validity of an early-stopping strategy based on validation-loss stagnation.

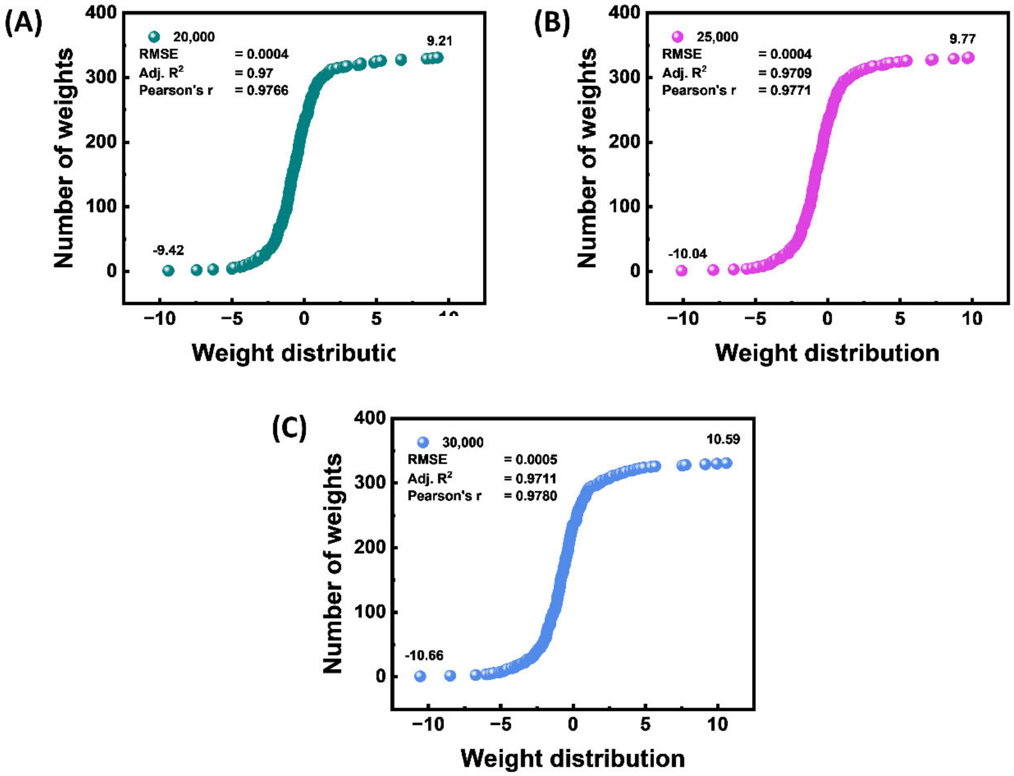

Figure S3. ANN weight distribution at different training stages. (**A**) 20,000, (**B**) 25,000, and (**C**) 30,000 iterations.

Figure S3 shows that the network has already converged architecturally, and thus functionally, long before these late checkpoints. It does this by tracking the distribution of connection weights after 20,000, 25,000, and 30,000 optimization steps. The cumulative curve in each panel maintains the same sigmoidal shape that initially emerged at approximately 5,000 iterations; only the extreme tails gradually move outward (from roughly ±9 at 20,000 to ±10 at 30,000), suggesting that a tiny percentage of already-dominant weights continue to grow while the great majority stay fixed. Figure S3A–C show that the inflection region of the curve (≆–1 to +3) remains unchanged, while the most extreme weights migrate slowly from ±9 to ±10.5; performance metrics stay statistically flat (Adj. $R^2 \approx 0.97 \pm 0.002$, RMSE ≈ $(4–5) \times 10^{-4}$), indicating that the model has already reached

its performance plateau. By increasing training to 30,000 iterations (Figure S3C), the tails are only elongated to ±10.5, but the RMSE, Adj. R^2 and Pearson's r improve by just 0.01%, 0.02%, and 0.1%, respectively. These findings confirm that structural convergence occurs before 20,000 iterations and that lengthy training primarily increases the size of saturated connections without improving generalization. This supports an early-stopping criterion of roughly 10,000–15,000 iterations for computational efficiency.

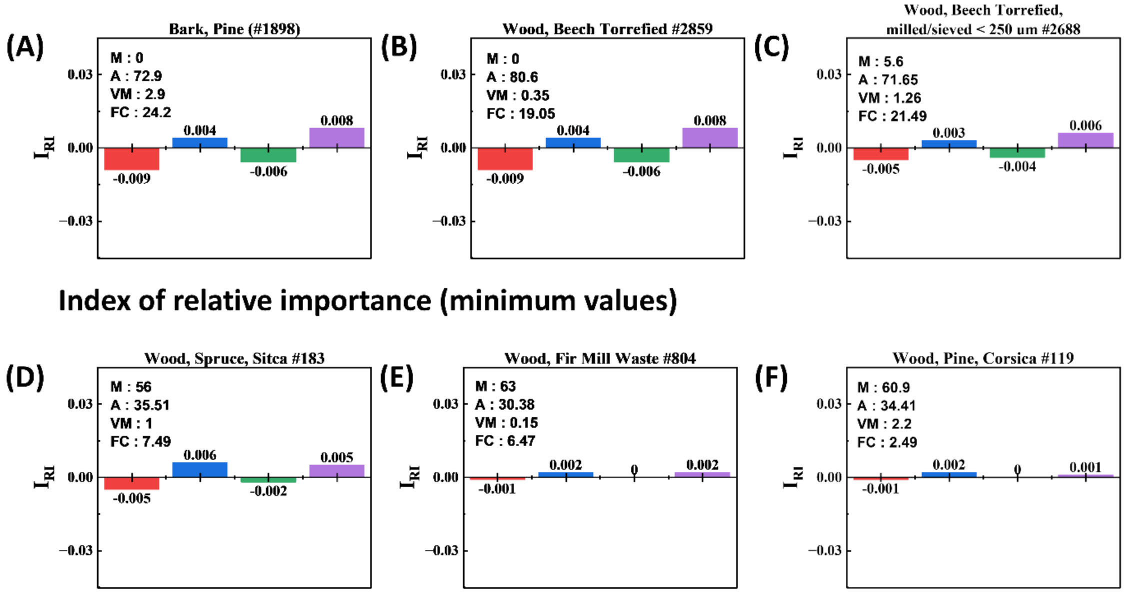

Figure S4. Relative importance index of physicochemical variables on (**A**–**C**) high values of higher heating value and (**D**–**F**) low values of higher heating value.

The Index of Relative Importance (IRI) for four important physicochemical variables: moisture (M), ash (A), volatile matter (VM), and fixed carbon (FC) on the prediction of Higher Heating Value (HHV) for various biomass types is shown in the picture. With positive or negative numbers signifying the direction and magnitude of each variable's influence on HHV, the IRI quantifies each variable's contribution. Figure S4A–S4C show the top three samples with the highest IRI values, indicating that input variables had the biggest impact on HHV prediction. Ash (A) and fixed carbon (FC) had the most significant effects in Figure S4A for Bark, Pine (#1898), for example, with FC contributing positively (IRI = 0.008) and ash negatively (IRI ≈ -0.009). Figure S4B and Figure S4C show similar patterns, with ash continuously showing a negative effect and fixed carbon a positive influence. This emphasizes FC as a major factor in increasing HHV. Samples with the lowest IRI values, where the factors have little impact on HHV, are shown in panels Figure S4D–Figure S4F. For instance, all the variables in Figure S4F (Wood, Pine, Corsica #119) have extremely low IRI values (around zero), suggesting that they have little to no effect on HHV for this biomass. Ash and moisture seem to have slight detrimental impacts, although fixed carbon continues to have a slight positive contribution.

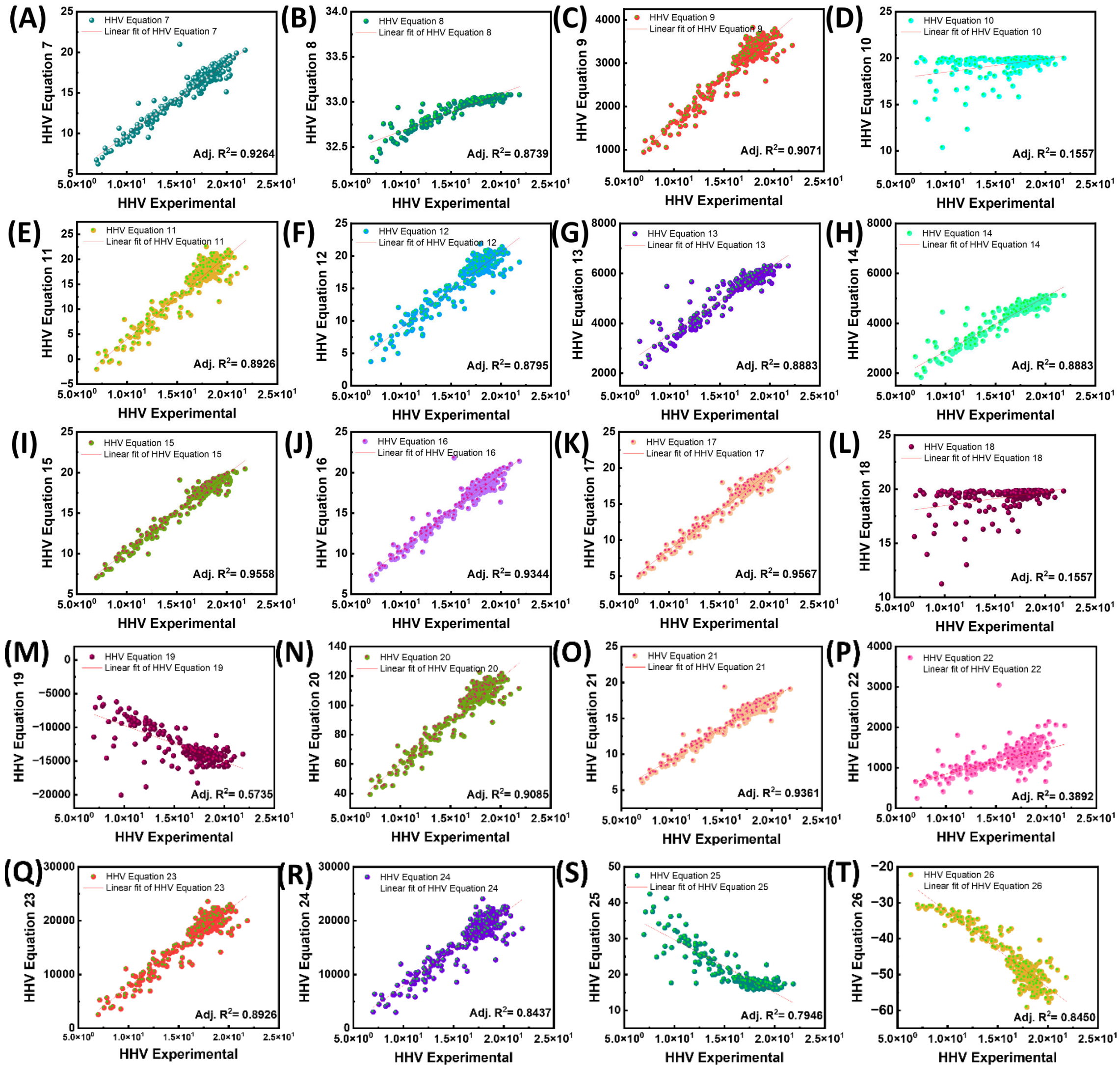

Figure S5. Comparison of experimental and predicted HHVs using proposed models: (**A**) Equation (7), (**B**) Equation (8), (**C**) Equation (9), (**D**) Equation (10), (**E**) Equation (11), (**F**) Equation (12), (**G**) Equation (13), (**H**) Equation (14), (**I**) Equation (15), (**J**) Equation (16), (**K**) Equation (17), (**L**) Equation (18), (**M**) Equation (19), (**N**) Equation (20), (**O**) Equation (21), (**P**) Equation (22), (**Q**) Equation (23), (**R**) Equation (24), (**S**) Equation (25), and (**T**) Equation (26).

A comprehensive comparison of presented and experimental Higher Heating Value (HHV) data using several suggested models is shown in the picture. The ideal 1:1 correlation is shown by the red line in each subplot (Figure S5A–S5T), which shows a scatter plot of projected HHV values vs experimental HHV values for a particular model. Each subplot provides the coefficient of determination (R^2) to measure the model's performance. Strong agreement between experimental and anticipated values is indicated by the R^2 values of 0.92635, 0.95581, and 0.95669 for equation. 7 (Figure S5A), equation 15 (Figure S5I), and equation 17(Figure S5K), respectively, demonstrating exceptional predictive skills. Similar to this, equation 9 (Figure S5C), equation 11 (Figure S5E), equation 12 (Figure S5F), equation 13 (Figure S5G), and equation 16 (Figure S5J) all show strong predictive performance, with R^2 values above 0.87. According to their reliability, the models equation 8 (Figure S5B) and equation 14 (Figure S5H) likewise display respectably high R^2 values (0.8739 and 0.88832, respectively). But the performance of equation 10 (Figure

S5D) and equation 18 (Figure S5L) is poor; both models produce low R^2 values of 0.15573, which show a substantial departure from the experimental data and a weak association.

The predictive performance also demonstrated by models like equation 20 (Figure S5N), equation 21 (Figure S5O), and equation 23 (Figure S5Q), exhibit a significant correlation between predicted and experimental data with R^2 values of 0.90849, 0.93611, and 0.89255, respectively. Additionally, the equation 24 (Figure S4R) model performs with an R^2 of 0.84359.

As evidenced by their low R^2 values of 0.57353, 0.38919, 0.9463 (with a negative trend), and 0.85404, respectively, other models, including equation 19 (Figure S5M), equation 22 (Figure S5P), equation 25 (Figure S5S), and equation 26 (Figure S5T), demonstrate poor agreement between experimental and projected values. Notably, equation 18 and equation 25 show negative trends, with projected values falling as experimental HHV rises. This suggests that the model is either underspecified or inappropriate for the dataset.

Table S1. The empirical formulas derived from the mentioned literature using data from proximate analysis.

Equation Number	Equations	Units	Ref.
(7)	HHV = 0.3536 × FC + 0.1559 × VM − 0.0078 × Ash	(MJ/kg)	[1]
(8)	$HHV = 33.078 - 0.72 \times (M/100) + 0.012 \times (M/100)^2 - 1.163 \times (M/100)^3 - 0.342 \times (Ash/100)^2$	(MJ/kg)	[2]
(9)	HHV = (44.75 × VM − 5.85 × M + 21.2)	(MJ/kg)	[3]
(10)	HHV = (20.086 − 0.261 × Ash)	(MJ/kg)	[4]
(11)	HHV = (13.173 + 0.416 × VM)	(MJ/kg)	[4]
(12)	HHV = 2.057 − 0.092 × Ash + 0.279 × VM	(MJ/kg)	[4]
(13)	HHV = 6295.7 − 64.05 × M	(MJ/kg)	[5]
(14)	HHV = 5112.0 − 52.08 × M	(MJ/kg)	[5]
(15)	HHV = 0.1905 × VM + 0.2521 × FC	(MJ/kg)	[6]
(16)	HHV = 0.3543 × FC + 0.1708 × VM	(MJ/kg)	[7]
(17)	HHV = 3.0368 + 0.2218 × VM + 0.2601 × FC	(MJ/kg)	[8]
(18)	HHV = 19.914 − 0.2324 × Ash	(MJ/kg)	[8]
(19)	HHV = 35.430 − 183.5 × VM − 354.3 × Ash	(MJ/kg)	[7]
(20)	HHV = 0.196 × FC + 14.119	(MJ/kg)	[9]
(21)	HHV = 0.312 × FC + 0.1534 × VM	(MJ/kg)	[9]
(22)	HHV = 82 × FC + a VM	(MJ/kg)	[10]
(23)	HHV = 356.248 × VM − 6998.497	(MJ/kg)	[11]
(24)	HHV = 356.047 × VM − 118.035 × FC − 5600.613	(MJ/kg)	[11]
(25)	$HHV = 75.56 - 1.3 \times (VM + Ash) + 7.03 \times 10^{-3} \times (VM + Ash)^2$	(MJ/kg)	[12]
(26)	$HHV = 4.183 \times 10^{-3} \times (8000 + VM) \times (70–1.65xVM)$	(MJ/kg)	[13]

HHV: Higher Heating Value; VM: Volatile Matter; FC: Fixed Carbon.

Table S2. Raw data used to generate the ANN proposed empirical equations.

	Sample	M	A	VM	FC	HHV
**1.**	**wood, beech torrefied at 260°C (#2860)**	0	0.4	75.7	23.9	**21.87**
**2.**	**wood, beech torrefied, milled/sieved < 250 um (#2688)**	5.6	1.26	71.65	21.49	**20.38**
**3.**	**wood, excess fraction wood from organic domestic waste compostin**	12.8	37.32	37.5	12.38	**9.7**
**4.**	**wood, excess fraction wood from organic domestic waste compostin**	9.9	29.73	45.5	14.87	**12.18**
**5.**	**wood, category B (painted) (#911)**	10.1	1.87	70.45	17.58	**17.85**
**6.**	**wood, demolition wood (#848)**	9.01	11.94	67.84	11.21	**16.75**

7.	wood, demolition wood (#2900)	15.3	6.18	63.27	15.25	**15.93**
8.	wood, demolition wood (#2901)	13	13.57	59.86	13.57	**14.77**
9.	wood, demolition wood, chipped (#676)	42.9	4.12	42.37	10.61	**10.81**
10.	wood, demolition wood, Fibra Flame pellets, Labee (NL) (#1370)	11.6	1.5	73.55	13.35	**17.54**
11.	demolition wood (#3498)	9.57	1.77	70.41	18.25	**18.26**
12.	shuttering wood (#1364)	33	6.27	48.64	12.09	**12.48**
13.	waste wood (#2748)	8.4	3.64	69.83	18.14	**17.77**
14.	wood, category B (painted), fine fraction (#938)	22.5	19.53	45.34	12.63	**12.01**
15.	railroad ties, sleepers (#654)	9.6	0.81	69.79	19.8	**19.16**
16.	railroad ties, sleepers (#885)	10.6	2.32	70.76	16.32	**18.86**
17.	urban waste wood (#1989)	14.64	2.72	63.7	18.93	**17.19**
18.	wood waste (#711)	9.28	6.58	67.61	16.53	**16.6**
19.	wood, furniture waste (#805)	12.07	3.17	72.98	11.77	**17.71**
20.	wood, waste wood (#2809)	33.1	0.54	54.62	11.75	**12.83**
21.	wood waste, dust (#655)	9.6	0.81	69.79	19.8	**17.9**
22.	wood, industrial wood waste, dust (#657)	11.3	0.35	71.85	16.5	**17.47**
23.	wood, chipped, >20 mm (#683)	12.4	2.54	67.19	17.87	**17.26**
24.	wood, chipped, 10-20 mm (#684)	15.1	2.46	65.29	17.15	**16.47**
25.	wood, chipped, not sieved (#682)	12.6	7.08	63.63	16.69	**16.26**
26.	bark (#1409)	10.9	2.05	69.59	17.46	**20.2**
27.	bark, hardwood (#53)	12.3	3.5	70.36	13.84	**17.15**
28.	bark, hardwood (#255)	16.92	7.78	62.85	12.45	**14.93**
29.	bark, hardwood (#257)	25.11	8.39	54.92	11.58	**13.47**
30.	bark, pine (#1268)	5	1.52	68.21	25.27	**20.3**
31.	bark, pine (#1718)	16.2	8.97	52.79	22.04	**16.37**
32.	bark, pine (#1898)	0	2.9	72.9	24.2	**21**
33.	Bark, Spruce (#3158)	5.25	2.22	71.25	21.28	**18.79**
34.	Eucalyptus bark (#2815)	12	4.22	68.73	15.05	**16.25**
35.	wood, beech (#1709)	19	0.83	68.77	11.4	**15.95**
36.	beech from Rettenmayer, milled/sieved <250 um (#2687)	9.6	0.91	74.76	14.73	**18.1**
37.	wood, beech chips, Rettenmayer (#2215)	10.2	0.93	74.53	14.33	**17.13**
38.	wood, birech (#76)	35.6	0.9	53.97	9.53	**12.95**
39.	pine sawdust (#136)	13.67	1.15	69.45	15.73	**17.93**
40.	pine sawdust (#146)	40	0.39	49.15	10.46	**12.58**
41.	pine sawdust (#152)	8.18	0.33	77.53	13.96	**18.81**
42.	Wood chips, whole tree, pine (#3154)	3.87	0.58	76.9	18.65	**20.08**
43.	wood, pine (#126)	10	0.21	77.13	12.66	**18.5**
44.	wood, pine (#127)	8.77	0.38	78.28	12.56	**18.7**
45.	wood, pine (#128)	7.57	0.95	75.64	15.84	**18.92**
46.	wood, pine (#130)	7.07	0.34	78.04	14.54	**19.06**

47.	**wood, pine (#131)**	6.61	0.35	78.93	14.1	**18.98**
48.	**wood, pine (#132)**	6.28	0.23	78.66	14.83	**18.96**
49.	**wood, pine (#134)**	7.71	0.42	80.36	11.51	**18.93**
50.	**wood, pine (#135)**	6.23	0.6	77.4	15.77	**19.1**
51.	**wood, pine (#138)**	8.01	0.21	78.84	12.94	**18.91**
52.	**wood, pine (#140)**	11.96	0.52	73.21	14.32	**18.16**
53.	**wood, pine (#141)**	12.41	0.29	73.48	13.82	**17.81**
54.	**wood, pine (#142)**	10.85	0.52	75.6	13.03	**18.31**
55.	**wood, pine (#144)**	9.7	0.12	76.79	13.39	**18.38**
56.	**wood, pine (#148)**	5.53	0.74	77.95	15.79	**19.28**
57.	**wood, pine (#151)**	50	1.5	36.5	12	**10.49**
58.	**wood, pine (#153)**	0	0.44	84.27	15.29	**20.13**
59.	**wood, pine (#154)**	4.63	0.38	80.46	14.52	**18.98**
60.	**wood, pine (#156)**	7.8	0.3	78.18	13.73	**19.29**
61.	**wood, pine (#157)**	5.82	0.07	80.38	13.73	**19.38**
62.	**wood, pine (#1860)**	10.3	5.74	74.27	9.69	**17.46**
63.	**wood, spruce, sitca (#181)**	51.9	1	40.4	6.7	**9.4**
64.	**wood, spruce, sitca (#183)**	56	1	35.51	7.49	**7.84**
65.	**wood, white fir (#2134)**	9.53	0.45	75.71	14.3	**17.8**
66.	**Forest residue chips, pine spruce (#3155)**	6.3	1.25	74.3	18.15	**19.37**
67.	**Forest residue chips, pine spruce (#3156)**	6.32	3.79	69.42	20.47	**19.24**
68.	**pine chips (#2784)**	9.3	0.27	76.64	13.79	**18.44**
69.	**Stump chips (#3172)**	8.15	2.38	75.64	13.83	**18.38**
70.	**Stump Chips (#3173)**	8.13	1.7	76.52	13.65	**18.72**
71.	**wood + bark, pine chips (#1269)**	6	0.38	76.61	17.01	**19.02**
72.	**wood, Jack pine waste (USA) (#880)**	40	1.3	44.6	14.1	**12.18**
73.	**wood, pine shavings (#2770)**	45	0.06	46.59	8.36	**10.7**
74.	**wood, pine, Canada (#2912)**	5.8	1.5	82.43	10.28	**18.46**
75.	**wood, pine, corsica (#119)**	60.9	2.2	34.41	2.49	**7.13**
76.	**Palm Leaves (#3350)**	33.03	5.9	52.94	8.13	**12.11**
77.	**wood, oak, red (#96)**	14.42	2.36	69.38	13.84	**16.37**
78.	**wood, oak, red (#97)**	10.79	1.81	71.79	15.61	**17.5**
79.	**wood, oak, red (#99)**	6.88	0.59	77.12	15.41	**18.2**
80.	**wood, oak, red (#100)**	14.76	0.88	69.28	15.08	**16.69**
81.	**wood, oak, swamp (#305)**	15	0.69	70.95	13.36	**16.81**
82.	**wood, oak, swamp (#307)**	5.5	0.94	79.69	13.87	**18.6**
83.	**wood, oak, green whole tree, chipped (#888)**	42	1.6	46.69	9.71	**11.25**
84.	**wood, oak, red, sawdust (#803)**	11.45	0.27	76.35	11.93	**17.25**
85.	**wood, pyrenean oak residues pellets (#2894)**	4.7	3.54	79.71	12.06	**18.23**
86.	**wood, Pyrenean oak, branches >70 mm diameter (#2854)**	41.27	1.22	48.01	9.5	**11.26**

87.	**wood, Pyrenean oak, branches 20 - 70 mm diameter (#2853)**	40.62	1.78	47.99	9.61	**11.42**
88.	**wood, Pyrenean oak, pellets from branches 20 - 70 mm (#2856)**	5.58	2.65	76.05	15.72	**18.12**
89.	**wood, Pyrenean oak, pellets from branches < 20 mm (#2855)**	8.37	3.04	72.67	15.92	**17.81**
90.	**wood, Pyrenean oak, pellets from branches > 70 mm (#2857)**	6.32	2.25	76.36	15.07	**18.08**
91.	**wood, birch + maple (#69)**	10	0.41	75.68	13.91	**17.73**
92.	**wood, birch + maple (#70)**	8.12	0.4	76.84	14.64	**18.2**
93.	**wood, birch + maple (#71)**	12.37	0.55	73.98	13.1	**16.92**
94.	**wood, birch + maple (#73)**	10	0.78	76.03	13.19	**17.72**
95.	**wood, briquettes, Ecoblok, Almelo, the Netherlands (#863)**	3.93	0.42	75.7	19.94	**19.61**
96.	**wood, briquettes, Ecoblok, Almelo, the Netherlands (#865)**	3.7	0.3	75.5	20.5	**19.55**
97.	**wood, briquettes, Ecoblok, Almelo, the Netherlands (#866)**	3.8	0.27	75.32	20.61	**19.68**
98.	**wood, eucalyptus (#699)**	9.34	0.48	78.52	11.66	**17.42**
99.	**wood, eucalyptus (#1781)**	10.5	0.59	73.3	15.61	**19.13**
100.	**wood, eucalyptus (#1782)**	10.6	0.69	73.93	14.78	**19.17**
101.	**wood, eucalyptus (#1785)**	9.8	1.89	73.42	14.89	**19.39**
102.	**wood, gum chips (#248)**	4.18	2.15	79.45	14.22	**18.69**
103.	**wood, gum chips (#249)**	7.17	1.19	77.19	14.45	**18.31**
104.	**wood, mixed hardwood chips (#251)**	4.7	0.58	80.2	14.51	**18.63**
105.	**wood, mixed hardwood chips (#252)**	4.42	0.56	81.43	13.58	**18.65**
106.	**wood, mixed hardwood chips (#253)**	5	0.49	81.1	13.4	**18.72**
107.	**wood, mixed hardwood chips (#259)**	5.5	1.14	79.19	14.17	**18.51**
108.	**wood, mixed hardwood chips (#260)**	5.74	1.19	79.37	13.71	**17.97**
109.	**wood, mixed hardwood chips (#261)**	4.59	0.43	81.67	13.31	**18.87**
110.	**wood, mixed hardwood chips (#273)**	5.31	0.9	77.55	16.24	**18.57**
111.	**wood, mixed hardwood chips (#275)**	8.15	1.35	74.83	15.67	**18.12**
112.	**wood, mixed hardwood chips (#277)**	20.2	1.67	63.96	14.17	**15.55**
113.	**wood, mixed hardwood chips (#279)**	28.9	0.6	58.72	11.78	**13.43**
114.	**wood, mixed hardwood chips (#281)**	29.9	0.37	57.59	12.14	**13.59**
115.	**wood, mixed hardwood chips (#282)**	36	0.58	59.37	4.05	**12.49**
116.	**wood, mixed hardwood chips (#283)**	25.2	0.94	62.7	11.16	**14.57**
117.	**wood, mixed hardwood chips (#284)**	17.2	0.63	69.45	12.72	**16.23**
118.	**wood, mixed hardwood chips (#285)**	30	0.38	58.68	10.94	**13.59**
119.	**wood, oak, birch, maple (#106)**	11.9	2.92	70.57	14.62	**16.95**
120.	**wood, oak, birch, maple (#107)**	4.3	1.52	78.47	15.7	**18.64**
121.	**wood, oak, birch, maple (#108)**	10.6	2.24	72.27	14.89	**17.3**
122.	**wood, oak, birch, maple (#109)**	11.8	1.22	71.87	15.11	**17.12**
123.	**wood, oak, birch, maple (#110)**	4.5	2.16	77.2	16.14	**18.04**
124.	**wood, oak, birch, maple (#111)**	12.2	1.37	71.43	15	**16.95**
125.	**wood, robinia (#1711)**	38.3	1.18	48.92	11.6	**11.49**
126.	**wood, robinia (#1713)**	34.2	1.26	52.77	11.77	**13.03**

127. wood, robinia (#1714)	38.8	2.75	46.57	11.87	**12.56**
128. Olive branches (#3347)	13.83	1.92	70.12	14.13	**16.3**
129. Orange tree pruning (#3349)	31.09	3.02	55.78	10.11	**12.46**
130. Vine shoot (#3351)	8.93	2.36	70.72	18	**17.13**
131. soft wood (#300)	30.5	1.3	55.6	12.6	**13.99**
132. soft wood (#302)	35	2.88	50.9	11.23	**12.06**
133. wood, larch (#265)	44.6	0.39	50.69	4.32	**9.74**
134. forest residue (#1995)	29.6	0.84	53.43	16.12	**12.74**
135. wood, sawdust (#2030)	34.93	0.69	55.03	9.35	**12.63**
136. wood chips (#2718)	5.1	6.55	71.84	16.51	**17.45**
137. wood pellets from Labee, The Netherlands (#2243)	7	0.2	76.45	16.35	**17.96**
138. wood pellets from Labee, The Netherlands (#2245)	11	0.57	72.71	15.72	**18.26**
139. wood, forest residue (#846)	48.91	2.03	42.1	6.96	**10.3**
140. wood, whole tree hogfuel (#263)	33.85	1.42	53.59	11.13	**13.03**
141. wood, whole tree hogfuel (#1895)	3	16.38	68.18	12.44	**17.33**
142. wood pellets (#2808)	8.7	0.46	74.4	16.44	**18.2**
143. wood pellets from Labee, brown (#2241)	9	1	69.89	20.11	**17.675**
144. wood (#901)	33.9	13.48	42.3	10.31	**11.1**
145. wood (#904)	43.3	7.03	39.46	10.21	**10.66**
146. wood (#907)	38.2	12.76	39.12	9.92	**12.17**
147. Wood chips from urban pruning (#3342)	11.08	4.42	70.41	14.09	**16.85**
148. wood, stems (#903)	39.8	1.02	48.04	11.14	**12.1**
149. wood, stems (#906)	39.3	0.85	49.47	10.38	**12.2**
150. wood, stems (#909)	43.5	1.75	44.13	10.62	**11.02**
151. sieved 0.075-0.09 mm (#923)	3.33	1.66	82.85	12.16	**20.37**
152. sieved 0.250-0.355 mm (#925)	3.1	1.24	80.58	15.07	**20.24**
153. wood, mixed (#900)	36.1	17.32	36.55	10.03	**8.95**
154. wood, park waste wood bio-dry (#1088)	14.8	15.58	51.86	17.76	**13.84**
155. wood, park wood, branches (#667)	39	5.07	43.74	12.19	**11.07**
156. wood, park wood, stems (#668)	39.6	13.97	36.54	9.89	**9.08**
157. wood, park wood, stems and branches (#669)	35	25.59	30.23	9.18	**8.24**
158. wood, coarse poplar (#845)	6.74	1.49	80.33	11.43	**17.66**
159. wood, hybrid poplar (#806)	6.89	2.51	78.97	11.63	**17.71**
160. wood, Gliricidia sepium (#2117)	31	0.69	53.82	14.49	**15.26**
161. wood, Gmelina arborea (#2119)	25.6	0.73	61.75	11.92	**15.37**
162. Leucaea wood chips (#2829)	24	1.9	63.23	10.87	**14.8**
163. wood, Leucaena leucocephala (#2121)	26.9	0.72	54.09	18.29	**16.1**
164. wood, salix (#1998)	11.4	1.68	73.18	13.73	**17.19**
165. wood, willow (#719)	10.23	0.85	76.52	12.4	**17.39**
166. wood, willow (#947)	11.1	1.87	69.88	17.16	**17.92**

167.	**wood, willow variety (#851)**	13.58	0.95	73.38	12.09	**16.73**
168.	**wood, willow variety (#852)**	9.71	1.08	74.99	14.22	**18.25**
169.	**wood, willow variety (#868)**	11.49	1.33	72.43	14.75	**17.41**
170.	**wood, willow variety (#869)**	9.98	1.54	74.01	14.47	**17.64**
171.	**wood, willow variety (#870)**	11.06	0.94	74.83	13.17	**17.22**
172.	**sieved 0.125-0.18 mm (#921)**	2.77	2.02	82.45	12.76	**18.92**
173.	**sieved 0.250-0.35 mm (#922)**	4.05	2.08	82.8	11.06	**19.99**
174.	**wood, cilinders 10*10 mm (diameter * length) (#1371)**	15	1.67	67.58	15.76	**16.24**
175.	**wood, small fraction, saw dust (smaller than 2*2 mm) (diameter * le**	10.5	2.51	70.08	16.92	**16.78**
176.	**wood, willow tops (#720)**	7.21	2.17	73.92	16.7	**18.37**
177.	**wood, willow, cilinders 10*40 mm (diameter * length) (#1367)**	10.6	1.88	70.89	16.63	**16.03**
178.	**wood, beech torrefied at 240°C (#2859)**	0	0.35	80.6	19.05	**20.71**
179.	**wood, excess fraction from composting plant (#908)**	50.3	10.01	30.91	8.78	**8.45**
180.	**wood, category B (painted) (#939)**	4.65	2.77	73.04	19.55	**18.59**
181.	**wood, demolition wood (#2712)**	8.6	1.92	70.65	18.83	**17.91**
182.	**wood, demolition wood (#2918)**	11.6	3.53	70.57	14.3	**17.28**
183.	**wood, demolition wood, chipped (#679)**	21.4	0.97	62.96	14.67	**15.56**
184.	**wood, demolition wood, Fibra Flame pellets, Labee (NL) (#1448)**	4.68	2.85	71.59	20.88	**18.57**
185.	**wood, demolition wood, dust (#678)**	11.5	16.26	56.55	15.69	**15.38**
186.	**urban waste wood (#2031)**	30.78	4.08	52.56	12.58	**13.45**
187.	**wood waste (#710)**	10.22	6.21	67.31	16.26	**17.05**
188.	**wood, waste wood (#926)**	9.64	2.2	81.15	7.02	**19.98**
189.	**sweep waste containing wood, paint, sand (#677)**	2.5	77.69	21.16	-1.35	**4.14**
190.	**wood, particle board (#656)**	8.1	0.55	71.41	19.94	**18.38**
191.	**wood, cca treated wood (#1721)**	13	0.87	70.56	15.57	**17.92**
192.	**bark, hardwood (#254)**	20.4	6.8	59.32	13.48	**14.44**
193.	**bark, hardwood (#256)**	20.46	8.46	58.51	12.58	**14.09**
194.	**bark, oak (#1899)**	0	5.3	76	18.7	**19.47**
195.	**bark, pine (#3157)**	4.74	1.64	69.54	24.08	**19.96**
196.	**wood, beech (#1365)**	15.2	0.51	71.97	12.32	**16.38**
197.	**wood, beech chips Rettenmayer (#2905)**	8.9	1.15	73.7	16.25	**17.52**
198.	**wood, beech for torrefaction (#2858)**	10	0.27	75.78	13.95	**17.04**
199.	**pine sawdust (#147)**	35	0.37	52.42	12.21	**13.76**
200.	**pine sawdust (#1267)**	6	0.09	78.11	15.79	**19.09**
201.	**wood, pine (#129)**	10.81	0.31	74.77	14.11	**18.47**
202.	**wood, pine (#133)**	6.19	0.63	77.96	15.23	**18.96**
203.	**wood, pine (#137)**	9.78	0.42	75.04	14.75	**18.61**
204.	**wood, pine (#143)**	5.92	0.33	79.39	14.36	**19.05**
205.	**wood, pine (#149)**	4	0.12	81.27	14.6	**19.41**
206.	**wood, pine (#155)**	5	0.42	80.12	14.46	**19.68**

**207.**	**wood, pine (#773)**	6.2	0.47	85.78	7.55	**17.92**
**208.**	**wood, spruce, sitca (#182)**	58.3	1.6	33.19	6.91	**7.94**
**209.**	**pine sawdust briquettes (#2291)**	9.8	1.82	51.87	36.51	**15.32**
**210.**	**stump chips (#3174)**	8.4	2.06	75.2	14.34	**18.55**
**211.**	**wood, fir mill waste (#804)**	63	0.15	30.38	6.47	**7.56**
**212.**	**wood, lodgepole pine (#124)**	51	2.3	29.69	17.01	**9.24**
**213.**	**wood, oak (#2148)**	5.3	0.3	80.02	14.38	**17.2**
**214.**	**wood, oak, red (#98)**	9	1.04	75.88	14.09	**18.05**
**215.**	**wood, oak, red (#101)**	15.69	1.02	59.37	23.92	**19.2**
**216.**	**wood, oak, swamp (#306)**	10	0.6	76.56	12.83	**17.69**
**217.**	**wood, Pyrenean oak, branches <20 mm diameter (#2852)**	42.21	2.34	44.76	10.69	**11.1**
**218.**	**wood, birch + maple (#68)**	10	0.32	75.59	14.09	**17.7**
**219.**	**wood, birch + maple (#72)**	3.65	0.48	81.42	14.45	**18.82**
**220.**	**wood, briquettes, Ecoblok, Almelo, the Netherlands (#693)**	4.3	0.67	74.93	20.1	**19.62**
**221.**	**wood, briquettes, Ecoblok, Almelo, the Netherlands (#864)**	3.09	0.47	76.27	20.18	**19.77**
**222.**	**wood, cherry (#229)**	26	1	60.35	12.65	**14.48**
**223.**	**wood, eucalyptus (#1270)**	4	0.77	77.18	18.05	**19.02**
**224.**	**wood, eucalyptus (#1861)**	8.6	2.74	81.44	7.22	**17.25**
**225.**	**wood, mixed hardwood chips (#250)**	3	0.9	80.91	15.19	**18.69**
**226.**	**wood, mixed hardwood chips (#258)**	4	0.64	81.85	13.51	**18.96**
**227.**	**wood, mixed hardwood chips (#272)**	10.75	1.54	72.45	15.26	**17.61**
**228.**	**wood, mixed hardwood chips (#274)**	5.31	1.03	77.92	15.74	**18.27**
**229.**	**wood, mixed hardwood chips (#278)**	28.3	0.31	59.89	11.5	**13.91**
**230.**	**wood, mixed hardwood chips (#280)**	48.3	0.74	42.83	8.13	**10.06**
**231.**	**wood, mixed hardwood chips (#1894)**	3.7	0.59	82.48	13.23	**18.74**
**232.**	**wood, robinia (#1712)**	38	2.02	48.23	11.76	**12.11**
**233.**	**Almond tree pruning (#3343)**	11.4	1.67	70	16.93	**16.11**
**234.**	**Profactus (NL) fuel pellets (#2768)**	8.65	0.44	72.99	17.92	**18.68**
**235.**	**soft wood (#303)**	37.3	2	39.19	21.51	**11.17**
**236.**	**wood, cedar chips (#2929)**	12.4	0.9	71.7	15	**18.17**
**237.**	**wood, christmas trees (#847)**	37.81	3.24	46.01	12.94	**13.03**
**238.**	**wood pellets from Labee, The Netherlands (#2244)**	7	0.1	75.8	17.1	**18.38**
**239.**	**wood, sawdust (#2785)**	8	1.36	77.12	13.51	**16.7**
**240.**	**wood, alder + fir sawdust (#807)**	52.63	1.96	36.27	9.15	**9.65**
**241.**	**wood (#905)**	41.1	2.95	44.82	11.13	**11.43**
**242.**	**wood, park waste wood (#912)**	5.15	3.06	73.58	18.21	**18.51**
**243.**	**sieved 0.125-0.18 mm (#924)**	0.57	1.12	82.9	15.41	**19.88**
**244.**	**wood, park wood (#665)**	47.1	18.52	26.77	7.62	**6.98**
**245.**	**wood, poplar (#700)**	4.8	1.16	80.99	13.05	**18.56**
**246.**	**Coconut trunk (#2828)**	49	5.87	36.72	8.42	**9.05**

**247.**	**wood, willow (#345)**	43.5	0.9	47.12	8.48	**10.48**
**248.**	**wood, willow (#2715)**	11.3	1.15	71.49	16.05	**16.94**
**249.**	**wood, willow variety (#867)**	12.68	1.48	71.74	14.09	**17.15**
**250.**	**Sieved 0.075- 0.09 mm (#920)**	3.79	2.81	80.81	12.58	**20.28**
**251.**	**Willow (#3159)**	2.39	1.15	77.99	18.47	**19.28**
**252.**	**wood, willow chips (#1091)**	3.45	1.55	77.98	17.01	**18.92**

References

1. Parikh, J.; Channiwala, S.A.; Ghosal, G.K. A correlation for calculating HHV from proximate analysis of solid fuels. *Fuel* **2005**, *84*, 487-494, doi:https://doi.org/10.1016/j.fuel.2004.10.010.
2. Akkaya, A.V. Proximate analysis based multiple regression models for higher heating value estimation of low rank coals. *Fuel Processing Technology* **2009**, *90*, 165–170. https://doi.org/10.1016/j.fuproc.2008.08.016.
3. Thipkhunthod, P.; Meeyoo, V.; Rangsunvigit, P.; Kitiyanan, B.; Siemanond, K.; Rirksomboon, T. Predicting the heating value of sewage sludges in Thailand from proximate and ultimate analyses. *Fuel* **2005**, *84*, 849–857. https://doi.org/10.1016/j.fuel.2005.01.003.
4. Callejón-Ferre, A.J.; Velázquez-Martí, B.; López-Martínez, J.A.; Manzano-Agugliaro, F. Greenhouse crop residues: Energy potential and models for the prediction of their higher heating value. *Renew. Sustain. Energy Rev.* **2011**, *15*, 948–955. https://doi.org/10.1016/j.rser.2010.11.012.
5. Demirbas, A.; Dincer, K. Modeling Higher Heating Values of Lignites. *Energy Sources Part A: Recovery Util. Environ. Eff.* **2008**, *30*, 969–974. https://doi.org/10.1080/15567030601082811.
6. Chang, Y.F.; Lin, C.J.; Chyan, J.M.; Chen, I.M.; Chang, J.E. Multiple regression models for the lower heating value of municipal solid waste in Taiwan. *J. Environ. Manag.* **2007**, *85*, 891–899. https://doi.org/10.1016/j.jenvman.2006.10.025.
7. Yin, C.Y. Prediction of higher heating values of biomass from proximate and ultimate analyses. *Fuel* **2011**, *90*, 1128–1132. https://doi.org/10.1016/j.fuel.2010.11.031.
8. Cordero, T.; Marquez, F.; Rodriguez-Mirasol, J.; Rodriguez, J.J. Predicting heating values of lignocellulosics and carbonaceous materials from proximate analysis. *Fuel* **2001**, *80*, 1567–1571. https://doi.org/10.1016/S0016-2361(01)00034-5.
9. Sheng, C.; Azevedo, J.L.T. Estimating the higher heating value of biomass fuels from basic analysis data. *Biomass Bioenergy* **2005**, *28*, 499–507. https://doi.org/10.1016/j.biombioe.2004.11.008.
10. Demirbaş, A. Calculation of higher heating values of biomass fuels. *Fuel* **1997**, *76*, 431–434. https://doi.org/10.1016/S0016-2361(97)85520-2.
11. Feng, Q.; Zhang, J.; Zhang, X.; Wen, S. Proximate analysis based prediction of gross calorific value of coals: A comparison of support vector machine, alternating conditional expectation and artificial neural network. *Fuel Process. Technol.* **2015**, *129*, 120–129. https://doi.org/10.1016/j.fuproc.2014.09.001.
12. Kathiravale, S.; Muhd Yunus, M.N.; Sopian, K.; Samsuddin, A.H.; Rahman, R.A. Modeling the heating value of Municipal Solid Waste. *Fuel* **2003**, *82*, 1119–1125. https://doi.org/10.1016/S0016-2361(03)00009-7.
13. Majumder, A.K.; Jain, R.; Banerjee, P.; Barnwal, J.P. Development of a new proximate analysis based correlation to predict calorific value of coal. *Fuel* **2008**, *87*, 3077–3081. https://doi.org/10.1016/j.fuel.2008.04.008.
